# Interactions between semaphorins and plexin–neuropilin receptor complexes in the membranes of live cells

**DOI:** 10.1016/j.jbc.2021.100965

**Published:** 2021-07-13

**Authors:** Shaun M. Christie, Jing Hao, Erin Tracy, Matthias Buck, Jennifer S. Yu, Adam W. Smith

**Affiliations:** 1 Department of Chemistry, University of Akron, Akron, Ohio, USA; 2 Department of Cancer Biology, Cleveland Clinic, Cleveland, Ohio, USA; 3 Department of Physiology and Biophysics, School of Medicine, Case Western Reserve University, Cleveland, Ohio, USA; 4 Department of Radiation Oncology, Cleveland Clinic, Cleveland, Ohio, USA; 5 Cleveland Clinic Lerner College of Medicine, Cleveland Clinic, Cleveland, Ohio, USA

**Keywords:** membrane biophysics, membrane protein, fluorescence correlation spectroscopy, protein–protein interaction, cancer, cell signaling, co-IP, co-immunoprecipitation, eGFP, enhanced GFP, *f*_c_, fraction of cross-correlation, FP, fluorescent protein, mCh, mCherry, Nrp1, neuropilin-1, PIE-FCCS, pulsed interleaved excitation fluorescence cross-correlation spectroscopy, VEGF, vascular endothelial growth factor

## Abstract

Signaling of semaphorin ligands *via* their plexin–neuropilin receptors is involved in tissue patterning in the developing embryo. These proteins play roles in cell migration and adhesion but are also important in disease etiology, including in cancer angiogenesis and metastasis. While some structures of the soluble domains of these receptors have been determined, the conformations of the full-length receptor complexes are just beginning to be elucidated, especially within the context of the plasma membrane. Pulsed-interleaved excitation fluorescence cross-correlation spectroscopy allows direct insight into the formation of protein–protein interactions in the membranes of live cells. Here, we investigated the homodimerization of neuropilin-1 (Nrp1), plexin A2, plexin A4, and plexin D1 using pulsed-interleaved excitation fluorescence cross-correlation spectroscopy. Consistent with previous studies, we found that Nrp1, plexin A2, and plexin A4 are present as dimers in the absence of exogenous ligand. Plexin D1, on the other hand, was monomeric under similar conditions, which had not been previously reported. We also found that plexin A2 and A4 assemble into a heteromeric complex. Stimulation with semaphorin 3A or semaphorin 3C neither disrupts nor enhances the dimerization of the receptors when expressed alone, suggesting that activation involves a conformational change rather than a shift in the monomer–dimer equilibrium. However, upon stimulation with semaphorin 3C, plexin D1 and Nrp1 form a heteromeric complex. This analysis of interactions provides a complementary approach to the existing structural and biochemical data that will aid in the development of new therapeutic strategies to target these receptors in cancer.

The semaphorins are a large family of secreted and transmembrane ligands that regulate cell morphology and motility during development in a broad range of tissues (1). About 20 members of this ligand family are found in vertebrates, where they are categorized by homology into classes 3 to 7 (2). The plexin family are type I transmembrane receptors and act as the main binding partner for these ligands at the cell membrane. Nine plexins are found in vertebrates, grouped in class A to D based on homology (2, 3). Secreted class 3 semaphorins require an additional receptor moiety, neuropilin-1 (Nrp1) or neuropilin-2, which have no intrinsic enzymatic activity, but create a holoreceptor complex with plexin to promote signaling (2, 4, 5, 6). Complex formation is a necessary part of their signaling activity, yet a profile of these interactions is still lacking because of the difficulty of working with membrane proteins in their native environment. Studies of plexin–neuropilin–semaphorin have mostly focused on biochemical data or structures of soluble domains of the proteins to establish protein–protein interactions, whereas receptor interaction in the live cell environment has not been fully explored (7). Using pulsed-interleaved excitation fluorescence cross-correlation spectroscopy (PIE-FCCS), we are able to complement the cellular data and other biophysical methods in order to understand a broader range of interactions and their likely role in plexin-mediated signaling (8).

Of the class 3 semaphorins, semaphorin 3A is the most well studied for its function as a chemorepellent in axon guidance and growth cone collapse, where deletion can lead to excessive axonal branching (9, 10, 11, 12, 13, 14). However, this ligand has broad expression across tissues where it plays multiple functional roles, such as vessel branching of the developing cardiovascular system, lungs, and kidneys (15, 16, 17, 18). Another class 3 semaphorin, semaphorin 3C, is expressed in the developing nervous system where it acts as a repulsive cue to guide tissue borders (19). Signaling by this ligand is also required for cardiovascular and lung development, with knockout mice, in some genetic backgrounds, unlikely to survive past the first few days (16, 20, 21, 22, 23).While these ligands are an integral part of development, changes to their expression can lead to disease states. Dysregulation of semaphorin 3A or semaphorin 3C has been implied for cardiovascular disease and various cancers (21, 24, 25, 26, 27).

Signaling is initiated when semaphorins bind to plexin receptors. Semaphorin 3C regulates downstream signaling in the presence of Nrp1 (20), plexin B1 (28), plexin A2, or plexin D1 (29). Various co-immunoprecipitation (co-IP) experiments have suggested the formation of complexes between multiple combinations of these receptors (30, 31, 32). However, co-IP may not be completely accurate because of the removal of proteins from the cell membrane environment and loss of inhibitory conformations (33). The goal of this work was to determine which receptors interact prior to and following semaphorin 3A and semaphorin 3C stimulation in the membrane of live cells using PIE-FCCS.

Quantifying the interactions between membrane proteins is experimentally challenging, and only a few of the plexins have been investigated with quantitative biophysical methods. Our laboratory first reported the ligand-independent homodimerization of plexin A4 using PIE-FCCS (34). In that study, we found that deletion of the sema domain abrogated homodimerization. Later, the structure of this interaction was resolved for plexin A4, as well as plexin A2 and plexin A1, by Kong *et al.* (35) using X-ray crystallography and verified with fluorescence lifetime imaging–FRET. Nrp1 and plexin A2 dimers have also been investigated with co-IP and quantitative FRET assays. These studies reported that Nrp1 forms small multimers in its basal state but transitions to dimers following ligand stimulation (36, 37). As noted, semaphorin 3A and semaphorin 3C require Nrp1 in order to induce signaling through complex formation with plexin receptors, such as with plexin A2 (32, 38). A 7.0 Å low–medium resolution structure for the tripartite interaction of semaphorin 3A, plexin A2, and Nrp1 extracellular domains has been solved where the complex suggests a 2:2:2 stoichiometry with Nrp1 acting as the bridge between semaphorin 3A and plexin A2 (39). A schematic for this type of signaling complex is shown in Figure 1. In addition, signal propagation through plexin A4–Nrp1 complexes is supported by cell collapse and alkaline phosphatase–binding assays (10, 40, 41).

**Figure 1:**
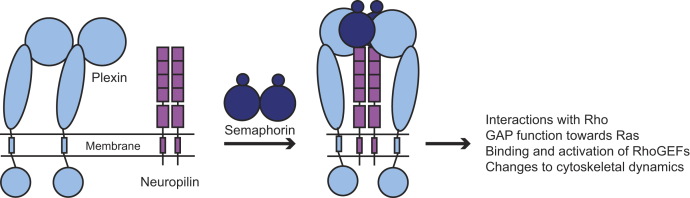
**Schematic of hypothesized plexin–neuropilin–class 3 semaphorin signaling.** Using previously available structural and biochemical data, plexins (*light blue*) and neuropilins (*purple*) likely form inhibitory homodimers. Addition of a soluble and dimeric class 3 semaphorin induces a tripartite complex formation where neuropilins act as a bridge between plexins and class 3 semaphorins. Conformational changes to the intracellular region of plexins then allow for interactions with GTPases, such as Rac1, R-Ras, and Rap1/2, which control downstream cytoskeletal dynamics.

In this study, we probed the interactions of Nrp1, plexin A2, plexin A4, and plexin D1 before and after stimulation with semaphorin 3A and semaphorin 3C using PIE-FCCS. Plexin D1 is activated by class 3 semaphorins but has not been investigated with cell biophysical assays. Thus, its oligomer state and potential heterotypic interactions have not been directly assessed. Our results confirm that in the absence of ligand, Nrp1, plexin A2, and plexin A4 each form homodimers. In contrast, we discovered that plexin D1 is monomeric. We also report here for the first time that plexin A2 and plexin A4 assemble into a heteromeric complex in the absence of ligand. Intriguingly, each of these homotypic dimer complexes (or lack thereof) was unaffected by semaphorin 3A or semaphorin 3C stimulation. A complex between plexin D1 and Nrp1 was observed following incubation with semaphorin 3C; however, no interactions were observed following semaphorin 3A stimulation. The results presented here expand upon previous interaction studies by including multiple receptor and ligand pairs to begin resolving the full interaction profile for this important protein family. Advances in understanding this local network of protein interactions will aid in the development of new therapeutic strategies that target these receptors.

## Results

### PIE-FCCS shows that Nrp1 forms multimers, plexin A2, and plexin A4 form homodimers, and plexin D1 does not self-associate

To measure the spatial organization of Nrp1, plexin A2, plexin A4, and plexin D1 in cells, we first expressed them individually by cotransfection of the enhanced GFP (eGFP) and mCherry (mCh) fusion constructs to determine their degree of homodimerization. PIE-FCCS data were collected from single live Cos-7 cells expressing the tagged protein of interest at surface densities ranging from 85 to 1245 molecules/μm^2^ (2) (sample data are shown in Fig. S3). From each cell measurement, we quantified expression of eGFP-labeled and mCh-labeled protein, the 2D mobility of the receptors in the plasma membrane, as well as the degree of association using the fraction of cross-correlation, *f*_c_ (8). In order to ensure that endogenous receptors would not interfere with the correlation analysis, we used Western blotting to confirm that transiently transfected plasmids are expressed at increased levels compared with endogenous expression (Fig. S1). For each protein we studied, the expressed protein band is larger than endogenous (control). The quantitative difference is not accessible by Western blot alone, and endogenous proteins will compete with expressed protein when there is dimerization. For this reason, the experimental *f*_c_ values should be thought of as a lower limit and could actually be higher in the absence of endogenous protein. PIE-FCCS does directly quantify the expression level of the fluorescent protein (FP) fusion in each single cell measurement, which varied between 85 and 1245 molecules/μm^2^ (2).

**Figure 2:**
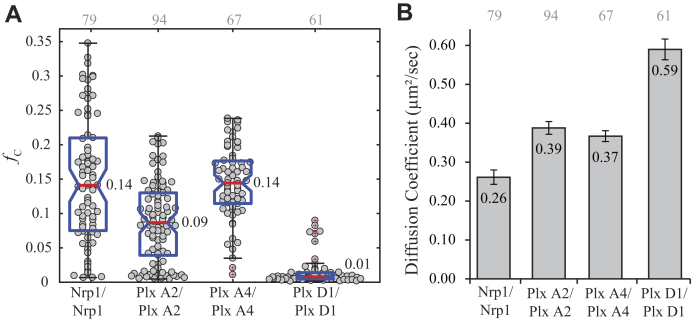
**Homotypic interaction of Nrp1, plexin A2, plexin A4, and plexin D1.***A*, fraction correlated for Nrp1, plexin A2, and plexin A4 fall in the range of homodimers, whereas plexin D1 diffuses as a monomer. *Gray* numbers above each column represent the number of single cells analyzed. *B*, the average diffusion coefficients agree with the cross-correlation results, where plexin D1 (monomer) diffuses at a faster rate than the dimers, but the slow diffusion for Nrp1 suggests that multimers may form as well, possibly involving interactions with other endogenous proteins. Nrp1, neuropilin-1.

The *f*_c_ values were used to determine the degree of oligomerization for each receptor (Fig. 2*A*). The Nrp1 data had a median of 0.14, which is consistent with strong dimerization; however, the wide distribution of *f*_c_ values over 0.20 suggests that Nrp1 can also form small homotypic multimers as reported previously (36, 37). The median *f*_c_ value of 0.14 for plexin A4 is consistent with dimerization, as reported previously by PIE-FCCS (34). The plexin A2 cross-correlation had a lower median (*f*_c_ = 0.09) indicating a weaker dimer affinity compared with plexin A4. To the best of our knowledge, there has been no investigation of the oligomerization state of plexin D1, except for a computational prediction that the isolated transmembrane helix is expected to dimerize to a similar extent as other plexin transmembrane domains (42). The near zero fraction correlated observed here (median *f*_c_ = 0.01) suggests that full-length plexin D1 does not dimerize in the given concentration range. The diffusion coefficients for each receptor support the interpretations of the *f*_c_ values (Fig. 2*B*), with higher mobility observed for monomeric plexin D1 compared with plexin A2 and plexin A4. Nrp1 has an average diffusion coefficient of 0.26 μm^2^/s, consistent with the formation of multimers as this is significantly slower than dimeric plexin A2 and plexin A4, where the average diffusion coefficients are 0.39 and 0.37 μm^2^/s, respectively. Plexin D1 has the fastest average diffusion coefficient, 0.59 μm^2^/s, adding to the evidence that it is monomeric.

### Nrp1 does not interact significantly with plexins in the absence of semaphorin ligand

**Figure 3:**
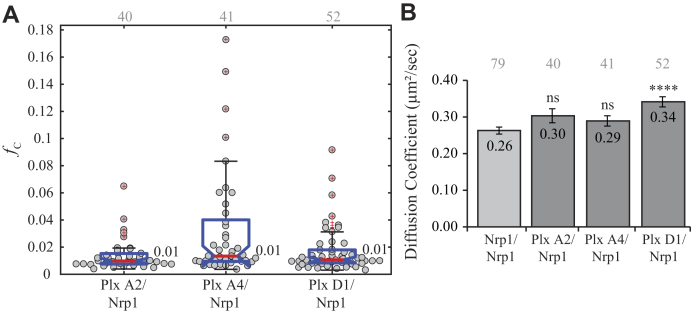
**Heterotypic interaction of Nrp1 with plexin receptors.***A*, the fraction correlated indicates no interaction between any receptor combinations under nonstimulatory conditions. *Gray* numbers above each column represent the number of single cells analyzed. Data marked with *red* + are regarded as outliers and are not included in the analysis. *B*, comparison of effective diffusion coefficient of Nrp1 when expressed alone (*light gray*) or coexpressed (*dark gray*). Comparison with Nrp1-mCh diffusion in the homodimer experiments shows that Nrp1-mCh diffusion is significantly increased when coexpressed with plexin D1 (*p* < 0.0001) but not with plexin A2 or plexin A4. mCh, mCherry; Nrp1, neuropilin-1.

Nrp1 is involved in class 3 semaphorin signaling as well as other ligands like vascular endothelial growth factor (VEGF) but cannot transduce the signal without expression of additional receptors (*i.e.*, plexins, VEGF receptor, mesenchymal–epithelial transition factor). However, conflicting reports exist regarding the interactions of these receptors in heteromeric complexes before and after stimulation. Man *et al.* (26) presented data suggesting the interaction of plexin A2, plexin D1, and Nrp1 following stimulation with semaphorin 3C, whereas others observed different heteromeric complexes prior to stimulation or even no interactions at all (20, 28, 30, 31). To measure the heterotypic interactions between Nrp1 and the plexin receptors, each plexin-eGFP construct was coexpressed with Nrp1-mCh. Single-cell PIE-FCCS data were collected for each combination to determine the degree of association and 2D mobility (sample data are shown in Fig. S4). The *f*_c_ values for Nrp1 coexpressed with each plexin construct each had a median value of 0.01 (Fig. 3*A*). This lack of cross-correlation indicates that unstimulated receptors have negligible propensity to dimerize with Nrp1 in the live cell plasma membrane. The average diffusion coefficient of Nrp1 expressed with plexin D1 showed a modest increase from 0.26 to 0.34 μm^2^/s compared with when it was expressed without plexin D1 (Fig. 3*B*). This suggests that Nrp1 may form oligomers when expressed alone but shifts toward dimerization when in the presence of coreceptors as previously reported (36, 37). The diffusion of the plexin receptors is not drastically altered in the presence of Nrp1 except in the case of plexin A4, which has a significant decrease in average diffusion coefficient, 0.37 to 0.31 μm^2^/s (Fig. S6). It is possible that plexin A4 interacts weakly with Nrp1 oligomers before stimulation, as evidenced by the comparatively large distribution of *f*_c_ values (Fig. 3*A*). With Nrp1 acting as a coreceptor for many other receptors (*e.g.*,VEGF receptor 2), the lack of cross-correlation in our assay may also be due to a competition between plexins and other endogenous receptors, that is, the plexin binding to Nrp1 is too weak to compete off these interactions.

### Class A plexins can form heterodimers *via* their sema domain, suggesting a heterotypic interaction model

Most studies on plexin–neuropilin–semaphorin signaling have focused on one receptor–ligand pair or the interaction with Nrp1. Therefore, little information has been reported for the heterotypic interactions of the plexins themselves. The previous report by Man *et al.* (26) suggested such interactions in glioblastoma multiform samples. However, because of the endogenous expression of semaphorin 3C, no unstimulated data were obtained. In a 2003 report, plexin A1 and plexin B1 were suggested to associate *via* their cytoplasmic domains (43). In another study, Smolkin *et al.* (29) determined that plexin A4 and plexin D1 were not associated when unstimulated but could form a complex when in the presence of semaphorin 3C.

**Figure 4:**
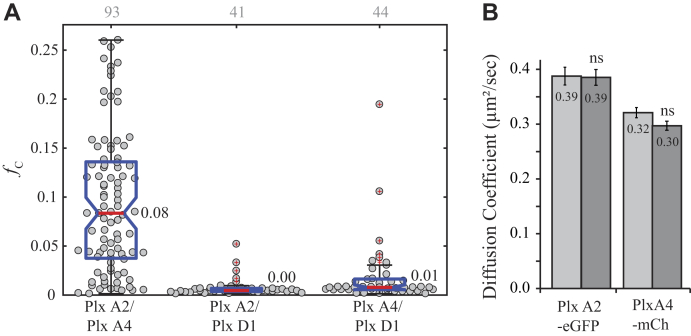
**Heterotypic interactions of plexins.***A*, fraction correlated for each combination indicates that plexin A2 and plexin A4 form a heterodimer, and neither interacts with plexin D1. *Gray* numbers above each column represent the number of single cells analyzed. *B*, diffusion coefficient change for plexin A2 and plexin A4 when expressed alone (*light gray*) or together (*dark gray*) likely indicating that neither forms a complex larger than a homodimer or a heterodimer.

To determine the degree of interaction between each plexin receptor pair, we conducted pairwise coexpression of each receptor combination in Cos-7 cells and collected PIE-FCCS data (sample data are shown in Fig. S5). The median *f*_c_ for plexin D1 coexpressed with either class A plexin is approximately zero (Fig. 4*A*). This indicates that neither class A plexin forms a complex with plexin D1 under nonstimulatory conditions. However, the median *f*_c_ value for plexin A2 and plexin A4 is 0.08, suggesting the presence of heterodimers in live cells. The average diffusion coefficient of both class A plexins is unchanged when expressed alone or with the other class A plexin (Fig. 4*B*). This allows us to conclude that the complex formed is most likely a heterodimer and not a larger multimer. This is the first observation, to the best of our knowledge, that class A plexins can be involved in heterodimers with other class A plexins prior to ligand stimulation.

Based on a crystal structure from Kong *et al.* (35), class A plexins form the inhibitory homodimer in a “head to stalk” fashion, with the sema domain acting as the “head” and the cysteine-rich plexin, semaphorin and integrin 2 domain and the immunoglobulin-like, plexin, transcription factors 2 domain acting as the “stalk” (cartoon is shown in Fig. 1). We coexpressed a mutant plexin A4 with a complete deletion of the sema domain (plexin A4ΔSema), previously used by Marita *et al.* (34) to determine if the heterodimer was also dependent on the sema domain. Using this construct in combination with WT plexin A2 shows a dramatic increase in the median *f*_c_ value, from 0.08 to 0.23 (Fig. S7*A*). The reason for the dramatic increase in cross-correlation is likely because of the reduced competition with the plexin A4 homodimers and possibly the release from an autoinhibited structure (see Discussion section). When plexin A4 lacks the sema domain, it can no longer form a homodimer, leading to the combinations A2:A2, A2:A4ΔSema, and an A4ΔSema monomer (Fig. S7*B*). The plexin A4 homodimer is now abrogated, reducing competition with the heterodimer, and causing the dramatic increase in codiffusion. These experiments show the necessity for heterodimerization analysis by PIE-FCCS to begin building a model that incorporates the full complexity of membrane protein interaction networks.

### Semaphorin 3C induces complex formation for plexin D1 and Nrp1, whereas semaphorin 3A does not induce a detectable interaction

**Figure 5:**
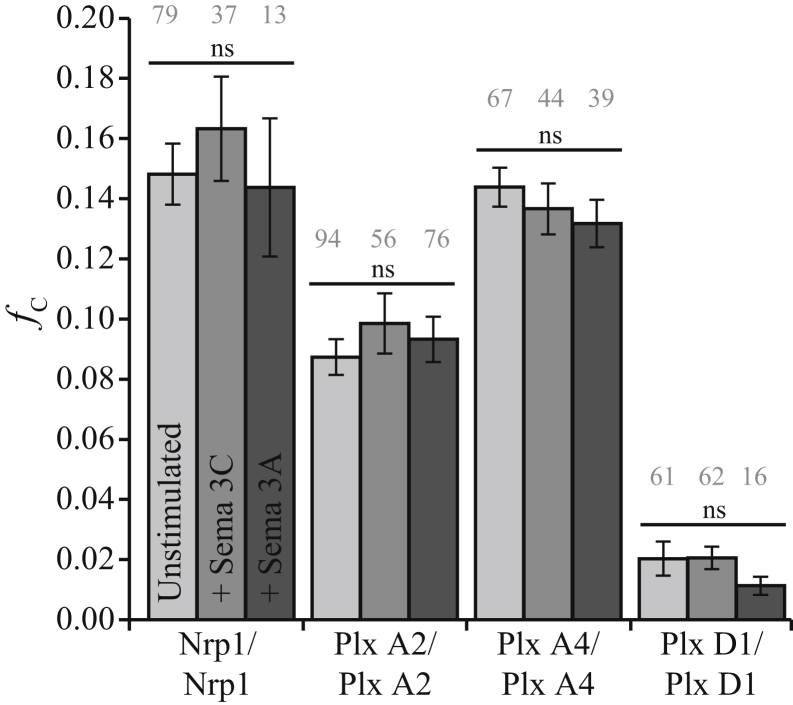
**Homotypic interaction of Nrp1, plexin A2, plexin A4, and plexin D1 following stimulation with semaphorin 3C and semaphorin 3A.** Cells expressing homotypic receptor combinations were incubated with 500 ng/ml of semaphorin 3C or semaphorin 3A 10 min prior to data acquisition. Fraction correlated is unchanged from nonstimulatory conditions. *Gray* numbers above each column represent the number of single cells analyzed. Nrp1, neuropilin-1.

Few data have been reported on whether the homotypic interaction of plexins is changed following ligand stimulation by semaphorin 3C or semaphorin 3A, except that direct binding to Nrp1 can occur (20, 31). Following the approach in the study by Man *et al.* (26), which delivers exogenous semaphorin 3C to cells in a dose-dependent manner, we incubated Cos-7 cells expressing individual receptors with 500 ng/ml of recombinant human semaphorin 3C or semaphorin 3A. Incubation times for previous experiments varied from minutes (29) to days (26) depending on the context of the experiment. Because we were interested in the early events of receptor interaction at the membrane rather than downstream signaling events, we collected PIE-FCCS data between 10 and 70 min after ligand stimulation. The average *f*_c_ value for each receptor was unchanged following stimulation indicating that homotypic oligomerization was not significantly enhanced or disrupted (Fig. 5). However, both plexin A4 and plexin D1 showed a significant increase in average diffusion coefficient following ligand stimulation, with semaphorin 3A (0.37–0.44 μm^2^/s) and semaphorin 3C (0.59–0.68 μm^2^/s), respectively (Fig. S8). These diffusion changes may be interpreted as a change in conformation and/or unbinding of an endogenous (unlabeled) protein, but it is unlikely that the homo-oligomerization state is affected by stimulation. This result is consistent with the crystal structure of the plexin A2–Nrp1–semaphorin 3A complex (35), where the interactions of Nrp1 are predominantly with the plexin A2–bridging semaphorin, with few or any Nrp1 domain contacts with plexin.

**Figure 6:**
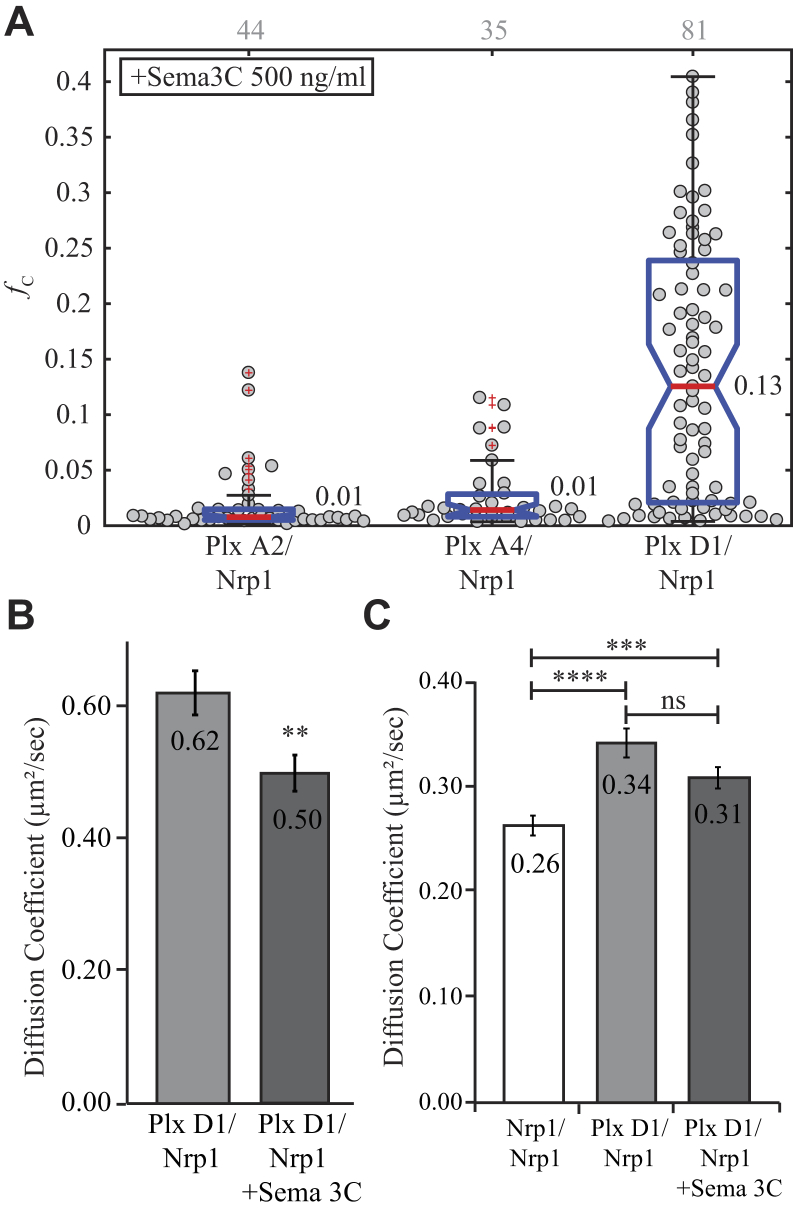
**Heterotypic interaction of Nrp1, plexin A2, plexin A4, and plexin D1 following stimulation with semaphorin 3C.***A*, fraction correlated for Nrp1 coexpressed with each plexin receptor. Plexin D1 and Nrp1 exhibit extensive dimerization. *Gray* numbers above each column represent the number of single cells analyzed. *B*, diffusion change for plexin D1-eGFP when coexpressed with Nrp1. Stimulation with semaphorin 3C significantly decreased (*p* < 0.01) the average diffusion coefficient indicating increased molecular weight and oligomer state. *C*, diffusion change for Nrp1-mCh alone or when coexpressed with plexin D1. Again, the average diffusion coefficient is significantly increased from expression alone but not significantly decreased from the unstimulated coexpression. eGFP, enhanced GFP; Nrp1, neuropilin-1.

We next tested the interaction between Nrp1 and each plexin receptor in the presence of semaphorin ligands. Each receptor combination was coexpressed in Cos-7 cells, and PIE-FCCS data were collected as in the previous experiments. Using the same concentration and incubation conditions from the previous section, the coexpressed receptors were stimulated with recombinant semaphorin 3C. The *f*_c_ values for Nrp1-mCh coexpressed with each plexin-eGFP construct are reported in Figure 6*A*. Plexin A2–Nrp1 and plexin A4–Nrp1 each have a median *f*_c_ value of 0.01, which is similar to the unstimulated values, indicating a lack of interaction. Both Man *et al.* (26) and Toyofuku *et al.* (32) observed plexin A2–Nrp1 Co-IP following semaphorin 3C stimulation, but analysis of PIE-FCCS data shows no interaction at this ligand concentration and receptor expression range (85–1245 molecules/μm^2^) in the live cell plasma membrane. Following semaphorin 3C stimulation, plexin D1–Nrp1 did show substantial increase in heterodimerization (*f*_c_ = 0.13). These changes in oligomerization state of plexin D1 and Nrp1 were supported by changes in the effective diffusion coefficient (Fig. 6, *B* and *C*). The average diffusion coefficient of plexin D1 decreased by 20% (0.62–0.50 μm^2^/s), indicating increased molecular weight and a shift from monomer to heteromeric complex (Fig. 6*B*). The Nrp1 diffusion coefficient was significantly higher than in the homodimer experiments (0.31 compared with 0.26 μm^2^/s) but does not significantly decrease upon stimulation with semaphorin 3C (0.34–0.31 μm^2^/s). This result is consistent with a shift from Nrp1 homomultimers to heteromeric complexes (Fig. 6*C*). Fig. S9 shows that the *f*_c_ distribution for each combination of plexin receptors was relatively unchanged following semaphorin 3C stimulation.

**Figure 7:**
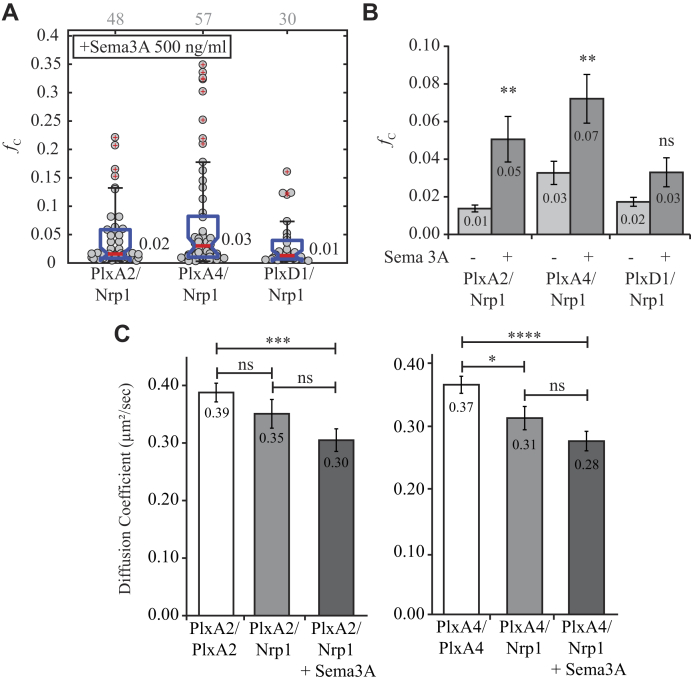
**Heterotypic interaction following semaphorin 3A stimulation.***A*, fraction correlated after stimulation. No apparent increase is observed like that of plexin D1–Nrp1 following semaphorin 3C stimulation. *Gray* numbers above each column represent the number of single cells analyzed. *B*, changes in average *f*_c_ value following stimulation. Plexin A2–Nrp1 and plexin A4–Nrp1 have significant increases in correlation, whereas the plexin D1–Nrp1 interaction is unchanged. *C*, diffusion change for plexin A4-eGFP and plexin A2-eGFP when coexpressed with Nrp1 and stimulated with semaphorin 3A. Both plexin A4 and plexin A2 have significantly decreased average diffusion coefficients adding to evidence that a weak/transient interaction is formed. *f*_c_, fraction of cross-correlation; Nrp1, neuropilin-1.

Various experiments have suggested that heteromeric complexes of Nrp1 and class A plexins form after stimulation (10, 12, 31, 38, 39, 40, 41, 44, 45, 46, 47). As demonstrated previously, before stimulation neither plexin A2 nor plexin A4 was observed to form a heteromeric complex with Nrp1. Using the same conditions as previously, Cos-7 cells coexpressing each combination of Nrp1 and plexin receptor were stimulated with semaphorin 3A and then probed with PIE-FCCS measurements to assess any changes in mobility and association. Figure 7*A* reports the *f*_c_ values for each set of receptors. No drastic changes in codiffusion were observed for any combination. In Figure 7*B*, we have compared the average fraction correlated for these combinations before and after stimulation and observed small but statistically significant increase for plexin A2–Nrp1 and plexin A4–Nrp1 when exposed to the ligand (0.01–0.05 and 0.03–0.07, respectively). Average diffusion coefficients for plexin D1 and Nrp1 were unchanged compared with homotypic and heterotypic interaction rates; however, plexin A4 and plexin A2 diffusion coefficients decreased (Fig. 7*C*). Both plexin A2-eGFP and plexin A4-eGFP have their lowest average diffusion coefficient when expressed with Nrp1 and stimulated with semaphorin 3A compared with expression of the plexin alone, 0.39 to 0.30 μm^2^/s and 0.37 to 0.28 μm^2^/s, respectively. Nrp1-mCh diffusion was not significantly changed under any condition. Overall, these data do not give any clear indication of a transition to heterodimer after semaphorin 3A binding as was seen for plexin D1 and Nrp1 following semaphorin 3C binding. However, some caution is advised when interpreting these negative results. PIE-FCCS measurements of heterodimers are affected by the stability and dynamics of the heterodimer as well as any competition with homodimers and heterotypic interactions with other endogenous receptors. Until the full network of membrane protein interactions can be resolved, it is difficult to rule out low-affinity interactions based on negative PIE-FCCS studies. Alternatively, it is also possible that three receptors may be necessary to form a stable signaling complex and that pairwise expression of exogenous receptors is insufficient to drive the formation of the full signaling complex. This type of heteromeric complex has been suggested in previous studies but not observed directly in live cell biophysical assays (10, 46, 48, 49). Future work using three-color labeling could help resolve these putative assemblies.

## Discussion

Semaphorin 3A and semaphorin 3C are needed for normal development, but disruption after the embryonic developmental stages can lead to various disease states. Depending on the tissue type, semaphorin 3A stimulation can enhance or inhibit angiogenesis and migratory pathways in tumor cell populations (6, 50, 51). Multiple studies have shown that reduction of semaphorin 3A expression occurs in later stages of cancers (including breast and prostate) and that exogenous semaphorin 3A leads to reduced metastasis and angiogenesis (27, 52, 53, 54, 55, 56, 57). Therefore, it is an important ligand to study as a potential antimigratory therapeutic factor (58). During development, semaphorin 3C downregulation in cardiac tissue is related to certain types of congenital heart disease (21). In later stages of life, semaphorin 3C overexpression is involved in multiple cancer types, including glioblastoma (26), lung (59), gastric (60), ovarian (61), and prostate (27, 28, 57). Angiogenesis can also be increased in the presence of semaphorins, and receptors for semaphorin 3C, particularly plexin D1, are upregulated in the tumor vasculature making it a potential drug candidate (30, 62). In order to fully understand the effects of these ligands, we must elucidate whether and how their receptors interact before stimulation and how their configurations are altered upon ligand–receptor complex formation. Previous work suggested that semaphorin 3C, plexin A2, plexin D1, and Nrp1 form a complex in glioma stem cells (26), whereas numerous studies have indicated the interaction of semaphorin 3A, plexins, and Nrp1. Our goal here was to determine the possible interaction modes in a live cell environment. Using PIE-FCCS, we were able to analyze these homotypic and heterotypic interactions of membrane receptors before and after ligand stimulation. Our work extends previous PIE-FCCS studies of plexin A4 dimerization to a larger set of receptors and ligands for which coexisting homodimers and heterodimers could compete for binding.

We first confirmed that Nrp1, plexin A2, and plexin A4, all form homodimers in the absence of ligand stimulation as previously reported (34, 35, 36, 37). We next determined that the full-length plexin D1 protein is a monomer, which to the best of our knowledge, is reported here for the first time. However, the homodimerization of plexin D1 was inferred based on computational prediction of reasonably strong interactions between the transmembrane helical domains (42). Different configurational states have been presented by crystallography and cryo-EM for the extracellular region of plexins over the last several years (35, 39), and the functional autoinhibition of such states can be relieved by truncation of the extracellular domains. For example, deletion of the plexin A1 sema domain converts the protein from an autoinhibited form to a constitutively active protein (in the absence of ligand) (47). In principle, it is possible that plexin D1 may undergo an inactive to active state transition without the need for homodimerization (7, 63, 64).

The receptors examined here do not form heterotypic interactions in the absence of ligand, except for plexin A2 and plexin A4. The class A plexins have conserved residues that may contribute to heterodimerization; however, these interactions had not been reported prior to the present study. Deletion of the sema domain from plexin A4 (plexin A4ΔSema) inhibits the homodimerization as we previously reported (34). When plexin A4ΔSema was coexpressed with WT plexin A2, there was a dramatic increase in the amount of cross-correlation and thus the degree of heterodimerization. This effect is ascribed to the fact that there was no longer competition from plexin A4 homodimers, allowing for a greater number of monomeric plexin A4ΔSema molecules to form A2:A4 heterodimers. The strong interaction between plexin A2 and plexin A4ΔSema also suggests that the dimerization is between the Sema domain of A2 and the “stalk” region of plexin A4ΔSema. This is consistent with the recent cryo-EM structures of the plexin ectodomains (35). These results support a model of heterotypic interactions where multiple binding partners and affinities must be taken into account to fully understand signaling. In addition, interactions such as these must be considered when disrupting or mutating receptors for disease-related research as signaling may still occur through related endogenous proteins.

**Figure 8:**
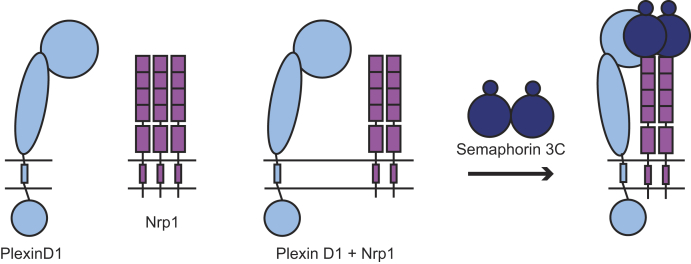
**Possible stoichiometry of plexin D1–Nrp1–semaphorin 3C complex.** Plexin D1 diffuses as a monomer, whereas initially Nrp1 diffuses as a dimer or a multimer. Upon coexpression, Nrp1 likely shifts toward dimers. Because of plexin D1 diffusing as a monomer, it is possible to form a 1:2:2 complex (*right*) using a plexin D1 monomer to induce signaling rather than a dimer seen for class A plexins. Nrp1, neuropilin-1.

After establishing the ligand-independent interactions, we now discuss the receptor interactions following stimulation with semaphorin ligands. PIE-FCCS shows that semaphorin 3C stimulation influences the interaction of plexin D1 and Nrp1, which was only indirectly observed in previous studies (20, 30). Our findings indicate that semaphorin 3C signal transduction may not utilize plexin A2 as a receptor, even though it appears to form a complex when observed by co-IP or alkaline phosphatase–binding assay (26, 32). Figure 8 shows a model of the plexin D1–Nrp1–semaphorin 3C interaction. Semaphorins are inherent dimers that have been shown to bind their receptors in a 2:2 stoichiometry as suggested by crystal structures (39, 65, 66). Taking this and our PIE-FCCS analysis into account, there are various interactions that may occur following semaphorin 3C stimulation. The first option is a 1:1:2 plexin D1–Nrp1–semaphorin 3C complex. Here, the median *f*_c_ value falls within the range expected for simple dimerization (67), but the values may be altered by monomeric plexin D1 and dimeric Nrp1. If the Nrp1 homodimer has a high binding affinity it is also possible that semaphorin 3C causes a 1:2:2 complex where a monomeric plexin D1 binds to a Nrp1 dimer upon stimulation (Fig. 8). In addition, plexin A2–Nrp1–semaphorin 3A form a 2:2:2 complex in the low/medium resolution crystal structure, and this receptor–ligand complex may have the same stoichiometry as shown in Figure 1 (35). Importantly, this Nrp1 domain only makes substantial contacts with the dimeric semaphorin 3A and not with the plexin A2 sema domain. Although the resolution of the complex structure was medium/low at 7 Å and Nrp1 domains a2, b1, and b2 were not seen in the crystal, the lack of Nrp1–plexin A2 interactions in the PIE-FCCS data is consistent with the negligible effect of ligand binding on plexin A2 and plexin A4 homodimerization. In the 2:2:2 crystallographic structure, there were no direct interactions between the plexin A2 sema domains. This is consistent with a model in which the sema-cysteine-rich plexin, semaphorin and integrin 2 domain and the immunoglobulin-like, plexin, transcription factors 2 domain interactions between plexins are replaced by sema domain interactions between plexin and semaphorin in the complex. Future experiments will need to be performed to determine the stoichiometry of receptors within the signaling complex as well as the time scales of the formation and disruption of the complex.

While previous reports have suggested that Nrp1 and class A plexins form a complex after stimulation with semaphorin 3A, our analysis by PIE-FCCS does not provide strong supporting evidence. A large increase in the *f*_c_ distribution, like that observed for plexin D1–Nrp1–semaphorin 3C, was not observed for Nrp1 and plexin A2 and A4 receptors when incubated with semaphorin 3A. There was a small but statistically significant increase in the mean *f*_c_ value for plexin A2–Nrp1 and plexin A4–Nrp1 with semaphorin 3A as well as a decrease in receptor mobility as seen in the diffusion coefficients (Fig. 7, *B* and *C*). There are several possible explanations for these results. First, the timescale of the association could be short. Our single cell measurements were performed during a period of 10 to 70 min after ligand addition. It could be that the formation of the complex is transient and thus appears weak in the average cross-correlation measurements. A second reason could be that there are multiple competing interactions with endogenous proteins. Because of the relative affinities of these competing interaction partners, there may be an ideal set of expression levels under which the heteromeric complex reported in the co-IP and crystallography studies is visible *via* PIE-FCCS. As stated previously, PIE-FCCS measurements of heterodimerization are affected by the stability and dynamics of the heterodimer as well as any competition with homodimers and heterotypic interactions with other endogenous receptors. Until the full network of membrane protein interactions can be resolved, it is difficult to rule out low-affinity interactions based on these negative PIE-FCCS results.

Finally, we report here that plexin A2 and plexin A4 form heterodimers, and that the extent of heterodimerization is unaffected by ligand binding. The observed A2/A4 heterodimer suggests that ligand binding induces a conformational change that activates the protein rather than driving dimerization *per se*. Studies of plexin B1 have led to a model in which pre-existing dimers are not just conformationally altered upon ligand binding, but that plexin–semaphorin 2:2 heterodimers may also associate to form larger order complexes (39). Our work suggests the possibility of a fundamental difference in the activation mechanism of plexin A, B, and D subfamilies.

One important consideration of any quantitative live cell biophysical method is the optimal concentration range of the experiment. For single-molecule imaging, this range is less than one molecule/μm^2^. For FRET experiments based on fluorescence intensity or lifetime, this range tends to be larger, typically from 10^2^ to 10^6^ molecules/μm^2^. The ideal range for PIE-FCCS is 10^1^ to 10^3^ molecule/μm^2^. For a commonly studied cell surface receptor, epidermal growth factor receptor, the protein density in normal cells is between 4 and 10 × 10^4^ molecules/cell (68) or between 60 and 140 molecules/μm^2^ (assuming a cell surface area of 700 μm^2^). We were not able to identify a report of the *in vivo* density of plexin receptors, but the expression is low in our model Cos-7 cells as seen in Fig. S1. The precise extent of dimerization *in vivo* will be dependent on cell type, expression level, and dimerization affinity. Future work will need to account for these parameters.

Overall, the work here has investigated only a subset of the potential interactions within the plexin–neuropilin–semaphorin protein family. More receptors and ligand combinations will need to be analyzed to establish a more expansive and holistic understanding of how membrane protein–protein interactions regulate plexin–semaphorin signaling. Because of the large number of ligands and receptors (seven class 3 semaphorins, nine plexin receptors, and two neuropilins), this is a time- and resource-intensive undertaking. The work we presented here lays the groundwork for such a comprehensive study. PIE-FCCS is an ideal method for quantifying these interactions in a live cell environment. Combined with cell signaling and high-resolution structure studies, it will be possible to resolve the function role of receptor homodimerization and heterodimerization in this important signaling axis. This work will also reveal how dysregulated signaling by plexins and neuropilins influence disease states, which will enable new approaches for designing therapeutic strategies.

## Experimental procedures

### Plasmids and cloning

Each of the full-length human receptor proteins were cloned into pEGFP-N1 and pmCh-N1 vectors for mammalian expression. Cloning of plexin A2-eGFP (accession no.: O75051) and plexin A2-mCh was carried out by inserting the plexin A2 sequence into EcoR1 and Kpn1 sites of the vectors. The cloning primers are

Forward: 5′ ACTGAATTCATGGAACAGAGGCGGCCCTGGCCCC 3′ and Reverse: 5′ ACTGGTACCGTGCTCTCAATGGACATGGCAT TAATGAGCTG 3′. Plexin D1-eGFP (accession no.: Q9Y4DY) and plexin D1-mCh were cloned by using EcoR1 and BamH1 sites in vectors and the plexin D1 internal Sac1 to amplify two pieces followed by a three-way ligation. The cloning primers are

Forward 1: 5′ AATGAATTCATGGCTCCTCGCGCCGCGGGCGGCGCACCCCTTAGCGCCCGGGCCGCCGCCGCCAGCCCCCCGCCGTTCCAGACGCCGCCGCGGTGCCCGGTGCCGCTGCTGTTGCTGCT 3′;

Reverse 1: 5′ GCACCAGGACCTGGAGCTCGGAGCCTACATGG 3′;

Forward 2: 5′ CCATGTAGGCTCCGAGCTCCAGGTCCTGGTGC 3′;

Reverse 2: 5′ AATGGATCCCGGGCCTCACTGTAGCACTCGTAGATGTTGTCCTCCATCAAAGCCAC 3′.

Cloning of Nrp1-eGFP (accession no.: O14786), Nrp1-mCh, plexin A4-eGFP (accession no.: Q9HCM2), plexin A4-mCh, plexin A4ΔSema-eGFP, and plexin A4ΔSema-mCh was performed as previously described (34). The plexin A4ΔSema mutant deletes residues 39 to 506 from the full-length construct near the N terminus.

### Cell culture and ligand stimulation

Cos-7 cells were cultured and transiently transfected using standard procedures (69). Briefly, culture media consisted of Dulbecco's modified Eagle's medium supplemented with 10% fetal bovine serum and 1% penicillin–streptomycin. Cells were passaged at 70 to 90% confluency to 35 mm glass bottom dishes (MatTek Corporation) for transfection. Approximately 24 h prior to data collection, the cells were transiently transfected with the protein(s) of interest using Lipofectamine 2000 reagent (Thermo Fisher Scientific) and 1.25 to 5 μg of plasmid DNA. Recombinant human semaphorin 3C (C636; Bon Opus Biosciences) contains residues 21 to 738 and is >95% pure. Recombinant human semaphorin 3A (CX65; Bon Opus Biosciences) contains residues 21 to 771 and is >95% pure. For stimulation with these ligands, a stock solution (100 μg/ml) was diluted to 500 ng/ml in imaging media and added to receptor-expressing cells approximately 10 min prior to data acquisition. Data were taken for up to 1 h following stimulation.

### PIE-FCCS instrumentation, data collection, and analysis

PIE-FCCS data collection was performed as previously described (8, 70). Briefly, the custom built setup uses a 50 ns pulsed continuum white laser source (SuperK Extreme; NKT Photonics) split into two wavelengths, 488 and 561 nm. These beams are directed through individual optical fibers of different lengths to induce a delay in arrival time relative to each other allowing for PIE and elimination of spectral crosstalk between the detectors (71). The beam powers were set to 300 nW for 488 nm and 800 nW for 561 nm. The beams were overlapped and directed to the back of the microscope (Eclipse Ti; Nikon Instruments). These overlapped beams were focused through the objective to a diffraction limited spot on a peripheral membrane area of a Cos-7 cell expressing the eGFP- and mCh-labeled receptor constructs. Emitted photons were detected by individual avalanche photodiodes with a 50 μm detection chip (Micro Photon Devices) and recorded by a time-correlated single-photon counting module running in time-tagged time-resolved mode.

For each single-cell measurement, five acquisitions of 10 s were recorded at the peripheral membrane area. Each intensity fluctuation was subjected to PIE gating before the autocorrelation and cross-correlation analysis. In an autocorrelation analysis, the intensity at time *F*(*t*) was compared with the intensity at a later time *F*(*t* + *τ*) and the self-similarity as a function of the later time allowed for interpretation of quantitative information such as diffusion and the number of particles. Intensity fluctuations were separated into 10 μs bins and subjected to the correlation algorithm in Equation 1, which normalizes the intensity change to the square of the average intensity (72, 73, 74). Cross-correlation uses the intensity fluctuations that occur simultaneously in both channels to infer interaction of species. Here, the correlation algorithm is represented by Equation 2 and the ratio of the cross-correlation amplitude to the autocorrelation amplitude indicates the proteins in complex, limited by the lower population molecule (72).

(1) $$G\left(\tau \right)=\left\langle F\left(t\right)F\left(t+\tau \right)\right\rangle /{\left\langle F\left(t\right)\right\rangle }^{2}$$

(2) $$G_{GR}\left(\tau \right)=\left\langle F_{G}\left(t\right)F_{R}\left(t+\tau \right)\right\rangle /\left\langle F_{G}\left(t\right)\right\rangle \left\langle F_{R}\left(t\right)\right\rangle$$

The five acquisitions from each single-cell measurement were averaged together to remove perturbations such as cell movement or bright clusters. Once individual curves from each cell are averaged, a least squares fitting to a 2D diffusion model is used, and includes fitting parameters for the triplet state (Equation 3).

(3) $$G\left(\tau \right)=\left(1+\frac{T}{1-T}e^{-\tau /\tau_{T}}\right)\left(\frac{1}{\left\langle N\right\rangle }\right)\left(\frac{1}{1+\tau /\tau_{D}}\right)+1$$

With autocorrelation, it is possible to infer protein diffusion by using the timing of intensity fluctuations. The half value decay time (lag time, $\tau_{D}$) can be used in Equation 4 to determine the effective diffusion coefficient (73). Diffusion will be affected by protein molecular weight and interactions with other molecules.

(4) $$D_{eff}=\frac{\omega^{2}}{4\tau_{D}*10^{-3}}$$

With the addition of a second detection channel, the codiffusion of two proteins can be analyzed by PIE-FCCS. The overlapping laser beams create a defined area for both eGFP- and mCh-tagged proteins, and their intensity fluctuations will occur simultaneously as they pass through the illuminated area (72, 73, 74, 75). Following cross-correlation by the algorithm stated previously, the amplitudes can be compared as shown in Equation 5 (72).

(5) $$f_{C}=\frac{{\left\langle N\right\rangle }_{gr}}{\min\left[\left({\left\langle N\right\rangle }_{gr}+{\left\langle N\right\rangle }_{r}\right),\left({\left\langle N\right\rangle }_{gr}+{\left\langle N\right\rangle }_{g}\right)\right]}$$

An ideal system would have a fraction correlated (*f*_c_) of zero for a noninteracting species and a fraction correlated of one for an interacting species. However, we must take certain considerations into account when interpreting live cell fluctuation results. Our laboratory's previous publications established a set of control constructs with a myristoylation anchor fused to dimerization motifs and an FP allowing for interpretation of *f*_c_ values for monomers, dimers, and higher order oligomers (Fig. S2) (8, 67). These constructs also indicate that the FPs themselves do neither cause nor inhibit dimerization. For homotypic interactions, median *f*_c_ values below 0.09 indicate monomeric species, 0.09 < *f*_c_ < 0.17 indicate dimeric species, and those above 0.17 indicate higher order oligomers.

### Western blotting

Samples for Nrp1, plexin A2, plexin A4, and plexin D1 endogenous expression in Cos-7 cells were collected 24 h after passaging. Samples for transient expression of Nrp1-eGFP, plexin A2-eGFP, plexin A4-eGFP, and plexin D1-eGFP in Cos-7 cells were transfected with 2.5 μg plasmid DNA 24 h after passaging and collected 24 h posttransfection. Cells were lysed using radioimmunoprecipitation assay lysis buffer supplemented with protease inhibitors (benzamidine, leupeptin, and PMSF). Western blotting experiments with these samples were carried out to confirm the expression of the samples. Primary antibodies used are Nrp1 (Cell Signaling; catalog no. 3725), plexin A2 (R&D; catalog no. MAB5486), plexin D1 (R&D; catalog no. AF4160), and plexin A4 (R&D; catalog no. MAB5856).

## Data availability

All data that support the findings of this study are contained within the article and its supporting information.

## Supporting information

This article contains supporting information.

## Conflict of interest

The authors declare that they have no conflicts of interest with the contents of this article.

## References

[1] Alto L.T., Terman J.R. (2017). Semaphorins and their signaling mechanisms. Methods Mol. Biol..

[2] Kruger R.P., Aurandt J., Guan K.L. (2005). Semaphorins command cells to move. Nat. Rev. Mol. Cell Biol..

[3] Junqueira Alves C., Yotoko K., Zou H., Friedel R.H. (2019). Origin and evolution of plexins, semaphorins, and met receptor tyrosine kinases. Sci. Rep..

[4] Pellet-Many C., Frankel P., Jia H., Zachary I. (2008). Neuropilins: Structure, function and role in disease. Biochem. J..

[5] Parker M.W., Guo H.F., Li X., Linkugel A.D., Vander Kooi C.W. (2012). Function of members of the neuropilin family as essential pleiotropic cell surface receptors. Biochemistry.

[6] Toledano S., Nir-Zvi I., Engelman R., Kessler O., Neufeld G. (2019). Class-3 semaphorins and their receptors: Potent multifunctional modulators of tumor progression. Int. J. Mol. Sci..

[7] Hota P.K., Buck M. (2012). Plexin structures are coming: Opportunities for multilevel investigations of semaphorin guidance receptors, their cell signaling mechanisms, and functions. Cell. Mol. Life Sci..

[8] Christie S., Shi X., Smith A.W. (2020). Resolving membrane protein-protein interactions in live cells with pulsed interleaved excitation fluorescence cross-correlation spectroscopy. Acc. Chem. Res..

[9] Klostermann A., Lohrum M., Adams R.H., Püschel A.W. (1998). The chemorepulsive activity of the axonal guidance signal semaphorin d requires dimerization. J. Biol. Chem..

[10] Yaron A., Huang P.H., Cheng H.J., Tessier-Lavigne M. (2005). Differential requirement for plexin-a3 and -a4 in mediating responses of sensory and sympathetic neurons to distinct class 3 semaphorins. Neuron.

[11] Eickholt B.J., Mackenzie S.L., Graham A., Walsh F.S., Doherty P. (1999). Evidence for collapsin-1 functioning in the control of neural crest migration in both trunk and hindbrain regions. Development.

[12] Nakamura F., Tanaka M., Takahashi T., Kalb R.G., Strittmatter S.M. (1998). Neuropilin-1 extracellular domains mediate semaphorin d/iii-induced growth cone collapse. Neuron.

[13] McCormick A.M., Jarmusik N.A., Leipzig N.D. (2015). Co-immobilization of semaphorin3A and nerve growth factor to guide and pattern axons. Acta Biomater..

[14] Taniguchi M., Yuasa S., Fujisawa H., Naruse I., Saga S., Mishina M., Yagi T. (1997). Disruption of semaphorin III/D gene causes severe abnormality in peripheral nerve projection. Neuron.

[15] Behar O., Golden J.A., Mashimo H., Schoen F.J., Fishman M.C. (1996). Semaphorin III is needed for normal patterning and growth of nerves, bones and heart. Nature.

[16] Kagoshima M., Ito T. (2001). Diverse gene expression and function of semaphorins in developing lung: Positive and negative regulatory roles of semaphorins in lung branching morphogenesis. Genes Cells.

[17] Reidy K., Tufro A. (2011). Semaphorins in kidney development and disease: Modulators of ureteric bud branching, vascular morphogenesis, and podocyte-endothelial crosstalk. Pediatr. Nephrol..

[18] Valdembri D., Regano D., Maione F., Giraudo E., Serini G. (2016). Class 3 semaphorins in cardiovascular development. Cell Adh. Migr..

[19] Steup A., Lohrum M., Hamscho N., Savaskan N.E., Ninnemann O., Nitsch R., Fujisawa H., Püschel A.W., Skutella T. (2000). Sema3C and netrin-1 differentially affect axon growth in the hippocampal formation. Mol. Cell. Neurosci..

[20] Gitler A.D., Lu M.M., Epstein J.A. (2004). Plexind1 and semaphorin signaling are required in endothelial cells for cardiovascular development. Dev. Cell.

[21] Feiner L., Webber A.L., Brown C.B., Lu M.M., Jia L., Feinstein P., Mombaerts P., Epstein J.A., Raper J.A. (2001). Targeted disruption of semaphorin 3C leads to persistent truncus arteriosus and aortic arch interruption. Development.

[22] Kodo K., Nishizawa T., Furutani M., Arai S., Yamamura E., Joo K., Takahashi T., Matsuoka R., Yamagishi H. (2009). Gata6 mutations cause human cardiac outflow tract defects by disrupting semaphorin-plexin signaling. Proc. Natl. Acad. Sci. U. S. A..

[23] Vadivel A., Alphonse R.S., Collins J.J., van Haaften T., O'Reilly M., Eaton F., Thébaud B. (2013). The axonal guidance cue semaphorin 3C contributes to alveolar growth and repair. PLoS One.

[24] Aggarwal P.K., Veron D., Thomas D.B., Siegel D., Moeckel G., Kashgarian M., Tufro A. (2015). Semaphorin3A promotes advanced diabetic nephropathy. Diabetes.

[25] Hu S., Zhu L. (2018). Semaphorins and their receptors: From axonal guidance to atherosclerosis. Front. Physiol..

[26] Man J., Shoemake J., Zhou W., Fang X., Wu Q., Rizzo A., Prayson R., Bao S., Rich J.N., Yu J.S. (2014). Sema3C promotes the survival and tumorigenicity of glioma stem cells through Rac1 activation. Cell Rep..

[27] Herman J.G., Meadows G.G. (2007). Increased class 3 semaphorin expression modulates the invasive and adhesive properties of prostate cancer cells. Int. J. Oncol..

[28] Peacock J.W., Takeuchi A., Hayashi N., Liu L., Tam K.J., Al Nakouzi N., Khazamipour N., Tombe T., Dejima T., Lee K.C., Shiota M., Thaper D., Lee W.C., Hui D.H., Kuruma H. (2018). Sema3C drives cancer growth by transactivating multiple receptor tyrosine kinases via plexin B1. EMBO Mol. Med..

[29] Smolkin T., Nir-Zvi I., Duvshani N., Mumblat Y., Kessler O., Neufeld G. (2018). Complexes of plexin-A4 and plexin-D1 convey semaphorin-3C signals to induce cytoskeletal collapse in the absence of neuropilins. J. Cell Sci..

[30] Yang W.J., Hu J., Uemura A., Tetzlaff F., Augustin H.G., Fischer A. (2015). Semaphorin-3C signals through neuropilin-1 and plexind1 receptors to inhibit pathological angiogenesis. EMBO Mol. Med..

[31] Rohm B., Ottemeyer A., Lohrum M., Püschel A.W. (2000). Plexin/neuropilin complexes mediate repulsion by the axonal guidance signal semaphorin 3A. Mech. Dev..

[32] Toyofuku T., Yoshida J., Sugimoto T., Yamamoto M., Makino N., Takamatsu H., Takegahara N., Suto F., Hori M., Fujisawa H., Kumanogoh A., Kikutani H. (2008). Repulsive and attractive semaphorins cooperate to direct the navigation of cardiac neural crest cells. Dev. Biol..

[33] Miernyk J.A., Thelen J.J. (2008). Biochemical approaches for discovering protein-protein interactions. Plant J..

[34] Marita M., Wang Y., Kaliszewski M.J., Skinner K.C., Comar W.D., Shi X., Dasari P., Zhang X., Smith A.W. (2015). Class a plexins are organized as preformed inactive dimers on the cell surface. Biophys. J..

[35] Kong Y., Janssen B.J., Malinauskas T., Vangoor V.R., Coles C.H., Kaufmann R., Ni T., Gilbert R.J., Padilla-Parra S., Pasterkamp R.J., Jones E.Y. (2016). Structural basis for plexin activation and regulation. Neuron.

[36] Chen H., He Z., Bagri A., Tessier-Lavigne M. (1998). Semaphorin-neuropilin interactions underlying sympathetic axon responses to class III semaphorins. Neuron.

[37] King C., Wirth D., Workman S., Hristova K. (2018). Interactions between NRP1 and VEGFR2 molecules in the plasma membrane. Biochim. Biophys. Acta Biomembr..

[38] Takahashi T., Fournier A., Nakamura F., Wang L.H., Murakami Y., Kalb R.G., Fujisawa H., Strittmatter S.M. (1999). Plexin-neuropilin-1 complexes form functional semaphorin-3A receptors. Cell.

[39] Janssen B.J., Malinauskas T., Weir G.A., Cader M.Z., Siebold C., Jones E.Y. (2012). Neuropilins lock secreted semaphorins onto plexins in a ternary signaling complex. Nat. Struct. Mol. Biol..

[40] Suto F., Murakami Y., Nakamura F., Goshima Y., Fujisawa H. (2003). Identification and characterization of a novel mouse plexin, plexin-A4. Mech. Dev..

[41] Casazza A., Laoui D., Wenes M., Rizzolio S., Bassani N., Mambretti M., Deschoemaeker S., Van Ginderachter J.A., Tamagnone L., Mazzone M. (2013). Impeding macrophage entry into hypoxic tumor areas by Sema3a/Nrp1 signaling blockade inhibits angiogenesis and restores antitumor immunity. Cancer Cell.

[42] Zhang L., Polyansky A., Buck M. (2015). Modeling transmembrane domain dimers/trimers of plexin receptors: Implications for mechanisms of signal transmission across the membrane. PLoS One.

[43] Usui H., Taniguchi M., Yokomizo T., Shimizu T. (2003). Plexin-A1 and plexin-B1 specifically interact at their cytoplasmic domains. Biochem. Biophys. Res. Commun..

[44] Takahashi T., Nakamura F., Jin Z., Kalb R.G., Strittmatter S.M. (1998). Semaphorins A and E act as antagonists of neuropilin-1 and agonists of neuropilin-2 receptors. Nat. Neurosci..

[45] De Wit J., De Winter F., Klooster J., Verhaagen J. (2005). Semaphorin 3A displays a punctate distribution on the surface of neuronal cells and interacts with proteoglycans in the extracellular matrix. Mol. Cell. Neurosci..

[46] Sabag A.D., Smolkin T., Mumblat Y., Ueffing M., Kessler O., Gloeckner C.J., Neufeld G. (2014). The role of the plexin-A2 receptor in Sema3a and Sema3b signal transduction. J. Cell Sci..

[47] Takahashi T., Strittmatter S.M. (2001). Plexina1 autoinhibition by the plexin sema domain. Neuron.

[48] Suto F., Ito K., Uemura M., Shimizu M., Shinkawa Y., Sanbo M., Shinoda T., Tsuboi M., Takashima S., Yagi T., Fujisawa H. (2005). Plexin-A4 mediates axon-repulsive activities of both secreted and transmembrane semaphorins and plays roles in nerve fiber guidance. J. Neurosci..

[49] Sharma A., Verhaagen J., Harvey A.R. (2012). Receptor complexes for each of the class 3 semaphorins. Front. Cell. Neurosci..

[50] Gu C., Giraudo E. (2013). The role of semaphorins and their receptors in vascular development and cancer. Exp. Cell Res..

[51] Bagci T., Wu J.K., Pfannl R., Ilag L.L., Jay D.G. (2009). Autocrine semaphorin 3A signaling promotes glioblastoma dispersal. Oncogene.

[52] Staton C.A., Shaw L.A., Valluru M., Hoh L., Koay I., Cross S.S., Reed M.W., Brown N.J. (2011). Expression of class 3 semaphorins and their receptors in human breast neoplasia. Histopathology.

[53] Pan H., Wanami L.S., Dissanayake T.R., Bachelder R.E. (2009). Autocrine semaphorin3A stimulates alpha2 beta1 integrin expression/function in breast tumor cells. Breast Cancer Res. Treat..

[54] Casazza A., Fu X., Johansson I., Capparuccia L., Andersson F., Giustacchini A., Squadrito M.L., Venneri M.A., Mazzone M., Larsson E., Carmeliet P., De Palma M., Naldini L., Tamagnone L., Rolny C. (2011). Systemic and targeted delivery of semaphorin 3A inhibits tumor angiogenesis and progression in mouse tumor models. Arterioscler. Thromb. Vasc. Biol..

[55] Chakraborty G., Kumar S., Mishra R., Patil T.V., Kundu G.C. (2012). Semaphorin 3A suppresses tumor growth and metastasis in mice melanoma model. PLoS One.

[56] Wang Z., Chen J., Zhang W., Zheng Y., Wang Z., Liu L., Wu H., Ye J., Zhang W., Qi B., Wu Y., Song X. (2016). Axon guidance molecule semaphorin3A is a novel tumor suppressor in head and neck squamous cell carcinoma. Oncotarget.

[57] Blanc V., Nariculam J., Munson P., Freeman A., Klocker H., Masters J., Williamson M. (2011). A role for class 3 semaphorins in prostate cancer. Prostate.

[58] Maione F., Capano S., Regano D., Zentilin L., Giacca M., Casanovas O., Bussolino F., Serini G., Giraudo E. (2012). Semaphorin 3A overcomes cancer hypoxia and metastatic dissemination induced by antiangiogenic treatment in mice. J. Clin. Invest..

[59] Rehman M., Tamagnone L. (2013). Semaphorins in cancer: Biological mechanisms and therapeutic approaches. Semin. Cell Dev. Biol..

[60] Miyato H., Tsuno N.H., Kitayama J. (2012). Semaphorin 3C is involved in the progression of gastric cancer. Cancer Sci..

[61] Capparuccia L., Tamagnone L. (2009). Semaphorin signaling in cancer cells and in cells of the tumor microenvironment--two sides of a coin. J. Cell Sci..

[62] Roodink I., Raats J., van der Zwaag B., Verrijp K., Kusters B., van Bokhoven H., Linkels M., de Waal R.M., Leenders W.P. (2005). Plexin D1 expression is induced on tumor vasculature and tumor cells: A novel target for diagnosis and therapy?. Cancer Res..

[63] Li Z.L., Müller-Greven J., Kim S., Tamagnone L., Buck M. (2021). Plexin-BS enhance their gap activity with a novel activation switch loop generating a cooperative enzyme. Cell. Mol. Life Sci..

[64] Mehta V., Pang K.L., Rozbesky D., Nather K., Keen A., Lachowski D., Kong Y., Karia D., Ameismeier M., Huang J., Fang Y., Del Rio Hernandez A., Reader J.S., Jones E.Y., Tzima E. (2020). The guidance receptor plexin D1 is a mechanosensor in endothelial cells. Nature.

[65] Antipenko A., Himanen J.P., van Leyen K., Nardi-Dei V., Lesniak J., Barton W.A., Rajashankar K.R., Lu M., Hoemme C., Püschel A.W., Nikolov D.B. (2003). Structure of the semaphorin-3A receptor binding module. Neuron.

[66] Love C.A., Harlos K., Mavaddat N., Davis S.J., Stuart D.I., Jones E.Y., Esnouf R.M. (2003). The ligand-binding face of the semaphorins revealed by the high-resolution crystal structure of Sema4d. Nat. Struct. Biol..

[67] Kaliszewski M.J., Shi X., Hou Y., Lingerak R., Kim S., Mallory P., Smith A.W. (2018). Quantifying membrane protein oligomerization with fluorescence cross-correlation spectroscopy. Methods.

[68] Carpenter G., Cohen S. (1979). Epidermal growth factor. Annu. Rev. Biochem..

[69] Aruffo A. (2002). Transient expression of proteins using cos cells. Curr. Protoc. Mol. Biol..

[70] Christie S.M., Ham T.R., Gilmore G.T., Toth P.D., Leipzig N.D., Smith A.W. (2020). Covalently immobilizing interferon-γ drives filopodia production through specific receptor-ligand interactions independently of canonical downstream signaling. Bioconjug. Chem..

[71] Müller B.K., Zaychikov E., Bräuchle C., Lamb D.C. (2005). Pulsed interleaved excitation. Biophys. J..

[72] Foo Y.H., Korzh V., Wohland T., Jung G. (2011). Fluorescence correlation and cross-correlation spectroscopy using fluorescent proteins for measurements of biomolecular processes in living organisms.

[73] Digman M.A., Gratton E. (2011). Lessons in fluctuation correlation spectroscopy. Annu. Rev. Phys. Chem..

[74] Briddon S.J., Kilpatrick L.E., Hill S.J. (2018). Studying gpcr pharmacology in membrane microdomains: Fluorescence correlation spectroscopy comes of age. Trends Pharmacol. Sci..

[75] Bacia K., Kim S.A., Schwille P. (2006). Fluorescence cross-correlation spectroscopy in living cells. Nat. Methods.

