# Supplementary material for: Interactions between semaphorins and plexin–neuropilin receptor complexes in the membranes of live cells
Source: J Biol Chem. 2021 Jul 13;297(2):100965. doi: 10.1016/j.jbc.2021.100965 (PMC8350011; doi:10.1016/j.jbc.2021.100965)
Supplement: Supplemental Figures S1–S10 [file mmc1.pdf]

Resolving the Interactions between Class 3 Semaphorin Receptors in Live Cells

Shaun M. Christie[1], Jing Hao[3], Erin Tracy[1], Matthias Buck[2], Jennifer S. Yu[3,4,5], Adam W. Smith[1]

[1]Department of Chemistry, University of Akron, Akron, OH 44325, USA

[2]Department of Physiology and Biophysics, Case Western Reserve University, School of Medicine, Cleveland, OH 44106, USA

[3]Department of Cancer Biology, Cleveland Clinic, Cleveland, OH 44195, USA

[4]Department of Radiation Oncology, Cleveland Clinic, Cleveland, OH 44195, USA

[5]Cleveland Clinic Lerner College of Medicine, Cleveland Clinic, Cleveland, OH 44195, USA

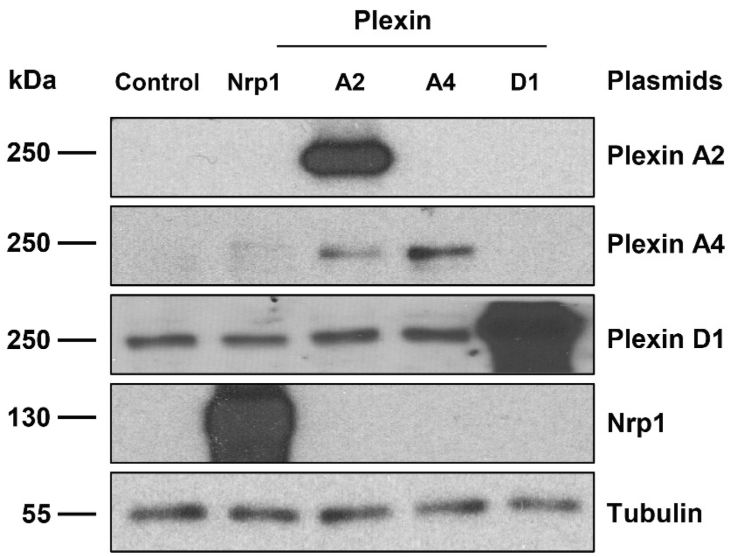

Figure S1. *Western blot of endogenous and transfected receptors. In each case of single transfection, transient receptors are expressed at higher levels than any endogenously present proteins. A band for transient Plexin A2 is present in the Plexin A4 transfected cells most likely due to non-specificity of the antibody.*

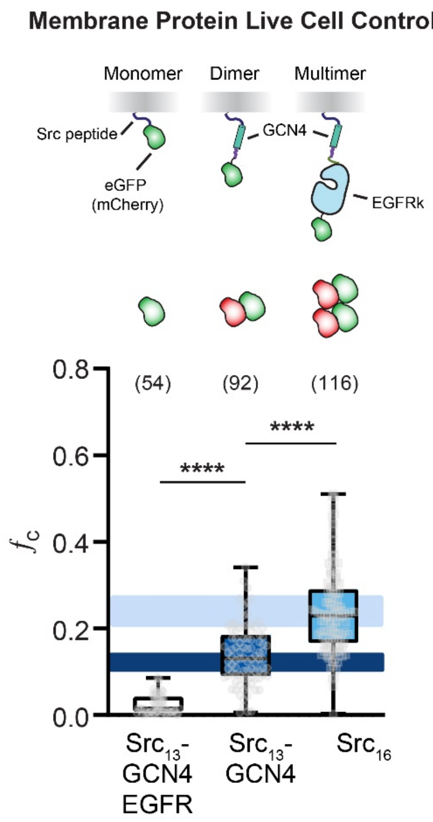

Figure S2. *Membrane protein controls for monomer, dimer, and multimer states. Top row shows schematics of the membrane protein chimeras. The first is a peptide with a myristoylation motif from the Src protein fused to a fluorescent protein (monomer, Src*$_{16}$*), the second has a GCN4 leucine zipper motif for dimerization (Src*$_{13}$*-GCN4), and the third has an additional kinase domain that drives multimer formation (Src*$_{13}$*-GCN4-EGFR). The distribution of single cell* f_c *values (gray markers) are overlayed with box and whisker plots for statistical comparisons.*

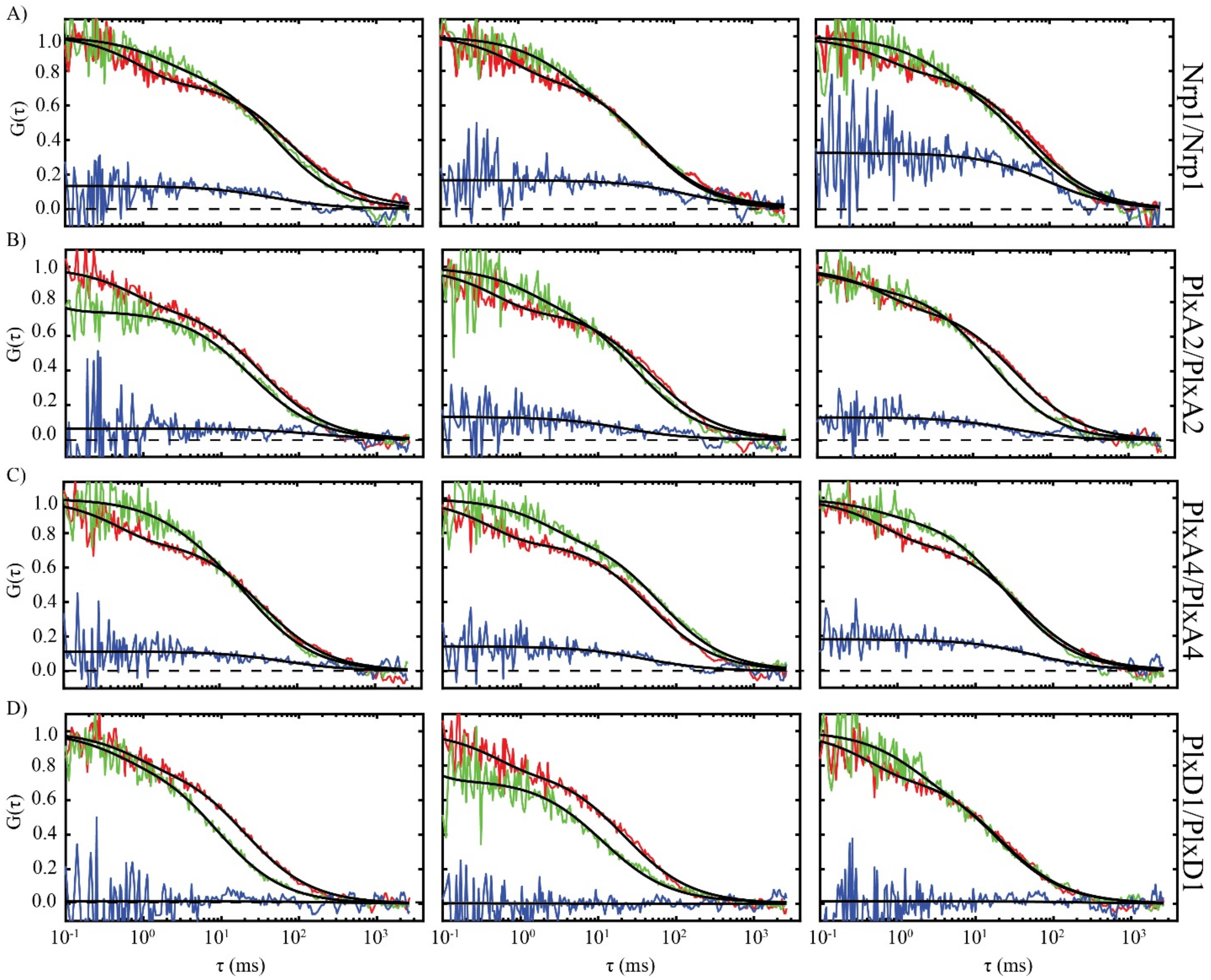

Figure S3. *Representative PIE-FCCS data for each homodimer experiment. Green and red lines are the autocorrelation functions (ACFs) obtained from fluorescence fluctuations in the green and red detection channels, respectively, while blue lines are the cross-correlation function (CCF) from the green and red co-diffusing species. The solid black lines are model fits used to calculate the density, diffusion coefficients, and fraction correlated (f_c) as described in the Methods section. The top row has three single cell data sets for homodimerization of Nrp1 (co-expression of Nrp1-eGFP and Nrp1-mCherry). The subsequent rows are for Plexin A2, Plexin A4, and Plexin D1.*

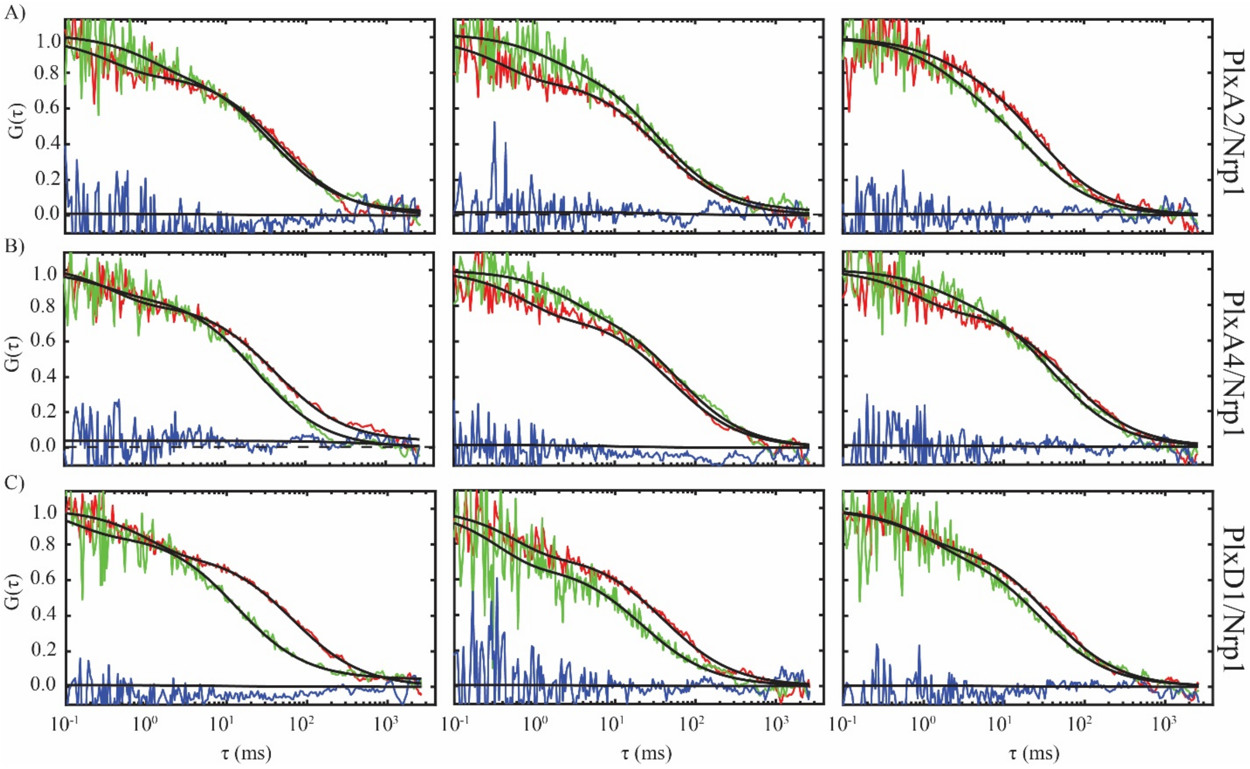

Figure S4. *Representative PIE-FCCS data for heterodimerization studies of Nrp1 with Plexin A2, A4, and D1. Green and red lines are the autocorrelation functions (ACFs) obtained from fluorescence fluctuations in the green and red detection channels, respectively, while blue lines are the cross-correlation function (CCF) from the green and red co-diffusing species. The solid black lines are model fits used to calculate the density, diffusion coefficients, and fraction correlated (f_c) as described in the Methods section.*

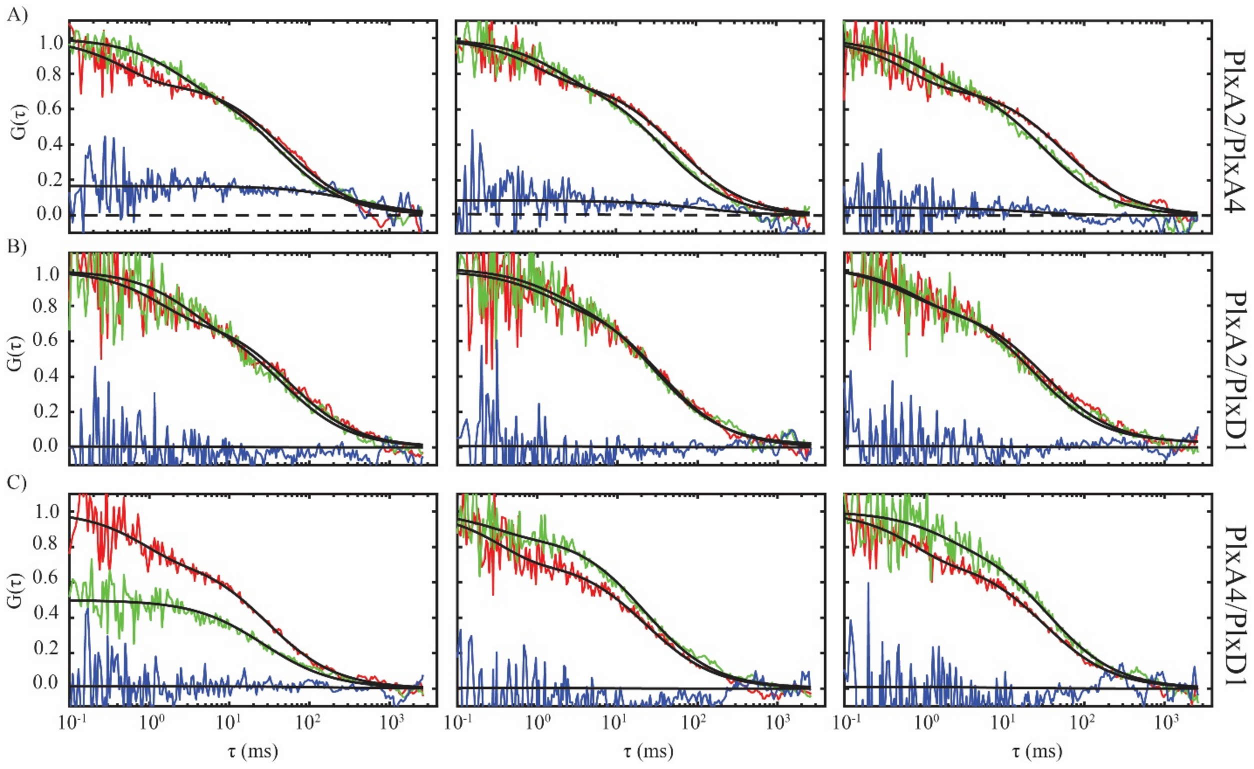

Figure S5. *Representative PIE-FCCS data for heterodimerization studies of Plexins A2, A4, and D1. Green and red lines are the autocorrelation functions (ACFs) obtained from fluorescence fluctuations in the green and red detection channels, respectively, while blue lines are the cross-correlation function (CCF) from the green and red co-diffusing species. The solid black lines are model fits used to calculate the density, diffusion coefficients, and fraction correlated (f_c) as described in the Methods section.*

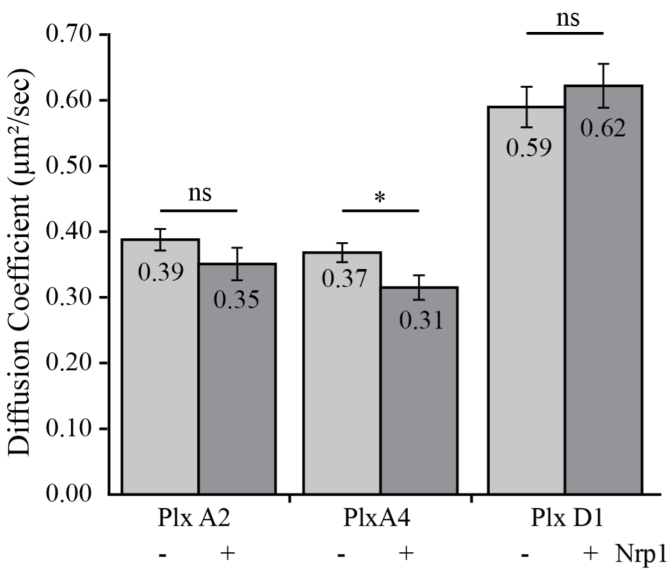

Figure S6. *Diffusion coefficients of plexins alone or co-expressed with Nrp1. Pairwise comparison of plexin-eGFP with and without co-expression of Nrp1 shows that only Plexin A4 has a significant decrease ($p<0.05$) in diffusion in the presence of Nrp1.*

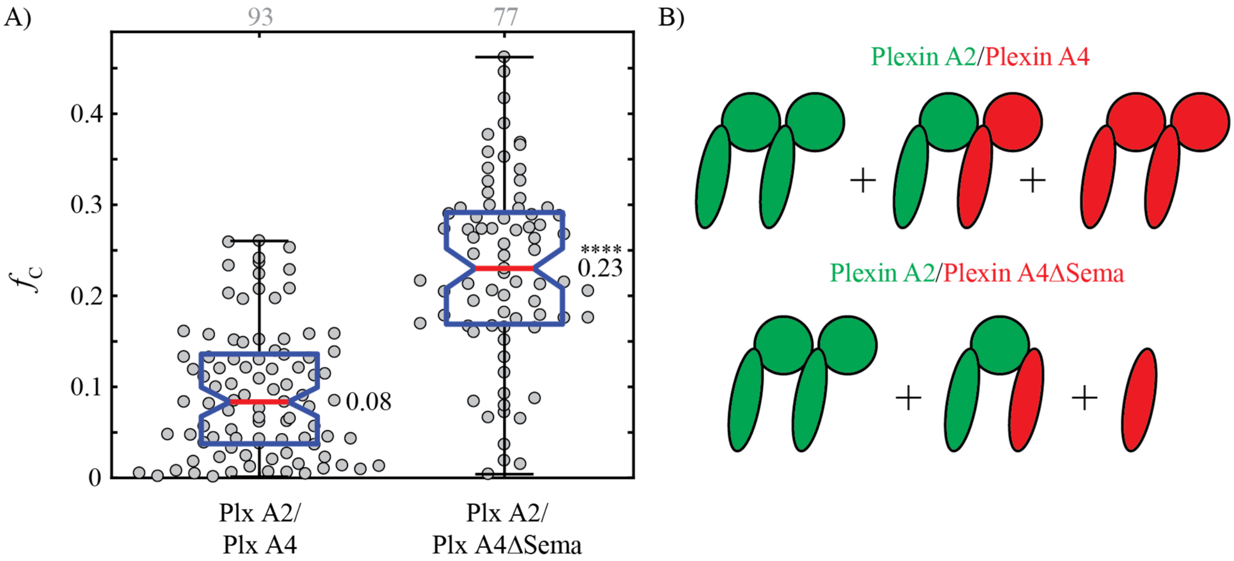

Figure S7. *Fraction correlated of class A plexin mutant variants. A) Plexin A4 has the entire Sema domain deleted and shows a dramatic increase in correlation with WT Plexin A2. Each combination is significantly different from each other (p<0.0001). Grey numbers above each column represent the number of single cells analyzed. B) Diagram of receptor combinations to show how interactions would change due to mutation. Top, homotypic and heterotypic affinities likely allow equal mixing of dimers and cause an* f_C *value associated with a weak dimer. Bottom, Removal of the Sema domain in Plexin A4 allows for a shift toward heterotypic interaction due to the lack of competition with homodimers of Plexin A4 leading to a drastic increase of the* f_C *value.*

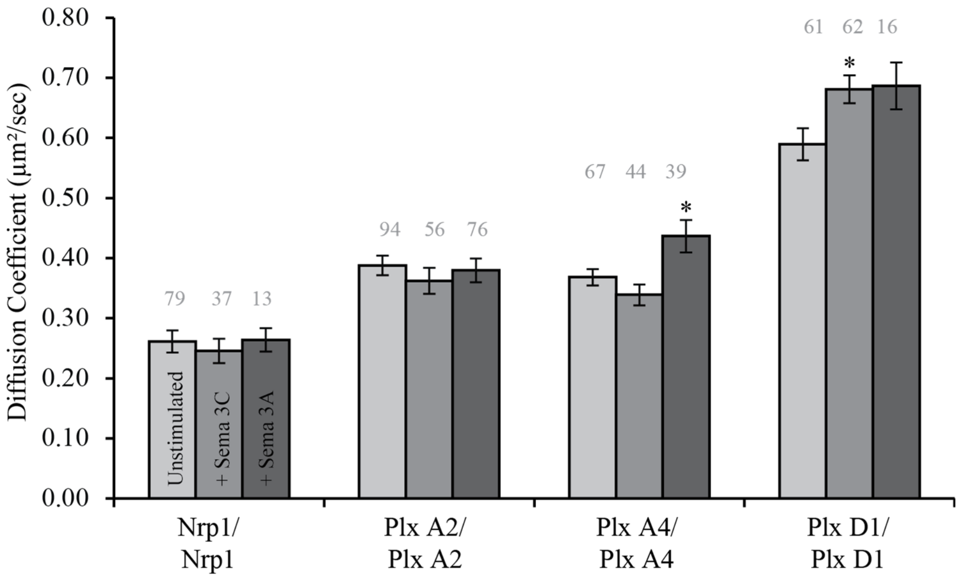

Figure S8. *Diffusion coefficients for Nrp1 and Plexin A2 homodimers are unchanged following stimulation with either ligand, while both Plexin A4 and Plexin D1 are significantly increased ($p<0.05$) for one type of ligand stimulation.*

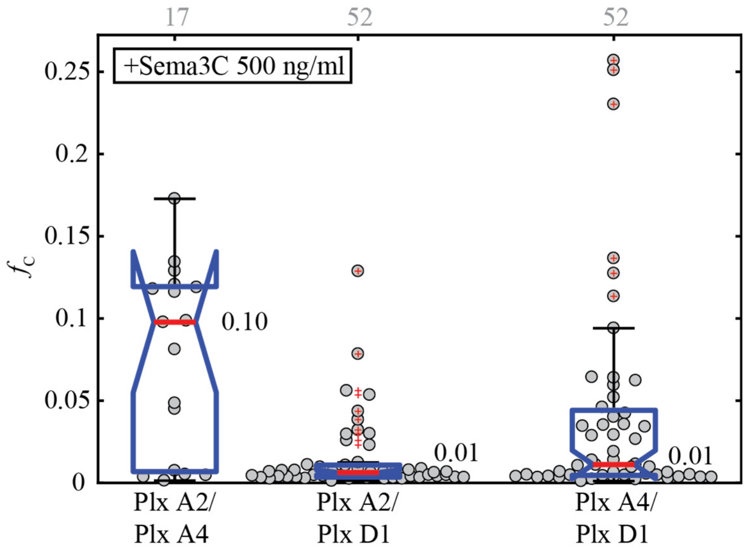

Figure S9. *Heterotypic interaction of plexins following stimulation with Semaphorin 3C. Fraction correlated for each combination of plexin receptors shows each interaction is not significantly changed from the unstimulated group.*

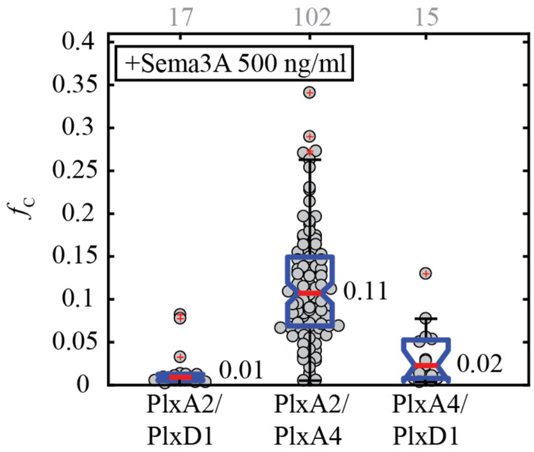

Figure S10. *Heterotypic interaction of plexins following stimulation with Semaphorin 3A. Fraction correlated for each combination of plexin receptors shows each interaction is not significantly changed from the unstimulated group.*
